# A Report of the Practice of Midwifery at the Westminster General Dispensary, during 1819

**Published:** 1822-05

**Authors:** 


Art. VII.—*A Report of the Practice of Midwifery at the Westminster General Dispensary, during* 1819. By the Editor.

[Concluded from page 288.]

II. *Classification of Miscarriages, according to their presumed Causes.*

TO ascertain, in a manner beyond a doubt, the immediate cause of abortions, is a point which I think worthy of some attention; and I therefore directed my inquiries towards this desirable end. In the success I have met with, I reckon as a favouring circumstance the retentive memory of mothers, who generally store up those events of their life to which I am now alluding, with a tenacity that nothing can relax. If the event itself be recollected, the causes which gave rise to it are also readily brought to memory, and accurately stated. Having found this to be the case, I can have no hesitation in giving the following statement as information which may be relied upon.

No attempt, that I am aware of, has ever been made to establish a methodical classification of miscarriages, and their causes; by which their study might be facilitated, and their treatment rendered more rational and successful. A gratuitous assumption on the part of some writers led them to adopt a division, first, into *miscarriages*, and, secondly, *premature labours* or *births*, according as the abortion takes place before or after the seventh month, from an idea that the fetus was susceptible of life at a certain period only. But the best authors have now rejected this distinction, as useless and incorrect. Abortion can mean nothing else than "the exclusion of the *ovum* from the uterus, before the ninth month." Children have come into the world at five and six months, who have lived. I have had an example in private, and another in my public, practice a few years back; and a number of similar cases are to be found in the older, as well as more recent, works on Midwifery. To apply, therefore, the name of *premature births* to cases of expulsion of the fetus after the seventh month, from a supposition that at that period alone the fetus is susceptible of life, is absurd. The fact is, that the propriety of producing abortion at the seventh and eighth months of pregnancy having been recommended, in some cases, by several very respectable writers and practitioners, it became absolutely necessary, in order not to shock the ears of the patient and those of her friends, to qualify the act by some plausible appellation; and this is the whole history of the invention of the words "premature labour."

In my Report published in 1819,* I proposed to arrange abortions, according to their presumed causes, into two distinct classes; to which if we refer the 372 miscarriages reported under the head G,† in the present Report, we shall find them to be thus distributed:

| | | | | |
|---|---|---|---|---|
| Class I. Constitutional. | 1. Active. | 1. Local or general fulness .......... | 67 | 77 |
| | | 2. Excessive irritability of the womb | 3 | |
| | | 3. Increased local action ............ | 7 | |
| | 2. Passive. | 1. Local or general debility .......... | 79 | 121 |
| | | 2. Certain morbid states of the body .. | 5 | |
| | | 3. Defective organization ............ | 3 | |
| | | 4. Peculiarities of the Ovum ........ | 9 | |
| | | 5. Habit and sympathy ............ | 25 | |
| | | | Total | 198 |

* See *A Report of the Practice of Midwifery at the Westminster General Dispensary, during* 1818. By A. B. Granville, M.D. &c. 1819.
† See *London Medical and Physical Journal*, No. 278, page 288.

| | | |
|---|---|---|
| | Brought up.................... | 198 |
| Class II. ACCIDENTAL. | 1. Fright .................................. | 80 |
| | 2. Falls .................................... | 22 |
| | 3. Violent exercise .......................... | 33 |
| | 4. Violent passions .......................... | 15 |
| | 5. Blows..................................... | 5 |
| | 6. Incautious use of medicines ................ | 3 |
| | 7. Improper moral and physical treatment of pregnant women .......................... | 12 |
| | * Not ascertained ........................... | 4 |
| | Total....— | 174 |
| | General Total .... | 372 |

**I. *Relative Periods of Pregnancy at which Abortion takes place.***

Next to the causes of miscarriage, I hold it to be an important object to know at what relative period of pregnancy this event generally occurs; and upon this subject I have obtained the information hereafter detailed, with regard to the 372 miscarriages which occurred to the 174 women before mentioned, during a period of ten years previous to 1819.

| | |
|---|---|
| At three months and under ---------- | 226 |
| From three to six months ------------- | 80 |
| From six to eight months ------------ | 66 |
| | 372 |

From this part of the report it will be an easy matter to deduce some curious calculations respecting the probabilities of a woman miscarrying at certain given periods of pregnancy. In my former report I have given a calculation of this kind, and established the chances of abortion taking place in any one of the nine months of pregnancy. The numbers quoted above do not lead to any very material difference in the results.

**K. *Number and Nature of Miscarriages, during* 1819, *at the Westminster General Dispensary.***

In the course of 1819, fifteen cases of either threatened or complete abortion came under my notice. Some of the former I was fortunate enough to prevent; and others were completed before I had it in my power to afford any assistance. I have always paid considerable attention to cases of abortion; because I think that much may be done to prevent them, when the first threatening symptoms make their appearance; nay, even when the process of abortion has actually begun: and also because, by watching narrowly the progressive course of a previous miscarriage, a recurrence of it may be averted. The importance of the subject has led me to keep a detailed account of those cases of abortion in which I had the means of trying the effect of care and medicines; and where I have failed, as well as where success has attended my attempts, I have noted in a book each particular case, as well as every circumstance which could lead to any important conclusion.

The following table contains an abstract of the fifteen cases in question.

| *Period of Pregnancy.* | *Names of Patients.* | | |
|---|---|---|---|
| At the third Month and under. | 1. Charlotte Smallwood | Completed before seen | Passive |
| | 2. Ann Thomson | Completed before seen | Passive |
| | 3. Sarah Thornton | Completed before seen | Accidental |
| .... | 4. Mary Dalerson | Completed before seen | Active |
| .... | 5. Eleanor Martin | Completed after being seen | Accidental |
| From the 3d to 6th Month. | 1. Ann Dudbridge | Completed after being seen | Passive |
| | 2. Mary Massey | Prevented—actually begun | Passive |
| .... | 3. Eliz. Chapple | Begun and effected, after being seen | Active |
| .... | 4. Martha Barrow | Prevented, though begun, after being seen | Passive |
| .... | 5. Martha Stubbs | Prevented—threatened, when called | Active |
| .... | 6. Eliz. Smart | Prevented, though begun, after being seen | Active |
| .... | 7. Rachel Wood | Effected before seen | Accidental |
| .... | 8. Jane Batts | Effected before seen | Passive |
| From the 6th to 8th Month, inclusive. | 1. Sarah Ashwood | Threatened, when seen, and prevented | Passive |
| | 2. M. Farrel | Effected before seen | Active |

The principal facts we collect from this table are these:—The proportion of abortions to the number of labours, during 1819, has been 1 in $45\frac{4}{5}$; and, with regard to the time at which they occurred, it appears to have been in the following ratio:

During the first period of pregnancy ------ 1 in 3,
— second period of pregnancy ---- 1 in $1\frac{7}{8}$,
— third period of pregnancy ------ 1 in $7\frac{1}{2}$.

If we look to the causes, we find the number of abortions produced by what are constitutional, and the purely accidental, to have been as 4 to 1.

It is also satisfactory to observe, that, whenever assistance was applied for in time, whether the miscarriage was merely threatening or had actually began, the success obtained from care and appropriate treatment has been considerable. Of eight such cases to which I was timely called, I succeeded in preventing abortion in five instances; although in three of them the process of abortion had actually began, and the flooding had been going on for some time, when I was first desired to attend.

My extended practice enables me to repeat the assertion, that almost every threatened miscarriage which has a constitutional cause for the origin, especially of the active kind, may be prevented.

In my former Report, I entered upon the consideration of the diseases of females, which, among the inferior classes of society, are unfortunately too frequent. Every privation they endure acts as an immediate cause of disease;—neglected suffering, of a trifling nature in the first instance, becomes often, with them, a more serious disorder;—and the incapacity, inattention, or presumptive interference, of a midwife or unskilled attendant, expose them, not unfrequently, to constitutional and sexual complaints, which might, otherwise, have never occurred.

The practice of the Westminster General Dispensary, in my department, has furnished me, during the year 1819, (of which I have been speaking in the present Report,) with an extensive number of examples illustrative of the foregoing observation. But, as it is not within my present plan to take a view of this subject, I shall defer the consideration of it to another opportunity. A. B. G.

---

